# Friction-lowering capabilities and human subject preferences for a hydrophilic surface coating on latex substrates: implications for increasing condom usage

**DOI:** 10.1098/rsos.180291

**Published:** 2018-10-17

**Authors:** Benjamin G. Cooper, Stacy L. Chin, Ruiqing Xiao, Karen Buch, Ducksoo Kim, Mark W. Grinstaff

**Affiliations:** 1 Department of Chemistry, Boston University, Boston, MA 02215, USA; 2 Department of Biomedical Engineering, Boston University, Boston, MA 02215, USA; 3 Department of Radiology, Boston University School of Medicine, Boston, MA 02118, USA; 4 Department of Medicine, Boston University School of Medicine, Boston, MA 02118, USA

**Keywords:** condoms, latex, lubricants, friction, touch

## Abstract

Personal lubricants can increase user satisfaction with male condoms by reducing friction and yielding a slippery sensation. However, lubricants pose disadvantages of dilution in physiologic fluids and sloughing away over repeated articulations. To address these drawbacks, a latex surface modification, which becomes lubricious in the presence of physiologic fluid, has been developed and evaluated. This study assesses (i) the frictional performance of the lubricious coating compared to non-coated latex and latex lubricated by personal lubricant, (ii) the level of agreement between human-perceived slipperiness and machine-measured friction, and (iii) human preference for a hypothetical male condom containing the lubricious coating. Friction coefficient of the lubricious coating was 53% lower than that of non-coated latex and approximately equal to that afforded by personal lubricant. A touch test and survey of a small population sample (*N* = 33) revealed a strong correlation (*R*^2^ = 0.83) between human-perceived slipperiness and machine-measured friction. A majority of participants (73%) expressed a preference for a condom containing the lubricious coating, agreeing that an inherently slippery condom that remained slippery for a long duration would increase their condom usage. Such a coating shows potential to be an effective strategy for decreasing friction-associated pain, increasing user satisfaction and increasing condom usage.

## 1. Introduction

The material surface properties of natural rubber latex male condoms are of significant importance during their use. Latex exhibits numerous advantages as the constituent material for male condoms, including excellent barrier properties, low manufacturing cost and facile processability. However, high surface friction at the latex-mucosa interface can cause a variety of problems including condom breakage [1], microtrauma to the mucosa [2] and discomfort [3–5]. As a result of the discomfort associated with condoms experienced by both men and women, along with their reported reduction in pleasure when used during intercourse [6] (noted by 77% of male and 40% of female respondents in a nationally representative 2008 study) [7], condoms may be used incorrectly or forgone altogether by some sexual partners, increasing the potential for unplanned pregnancy and transmission of STIs [8–12].

To address the limitations of high condom friction and discomfort, various personal lubricants are used among partners. Such lubricants, typically water-, silicone- or oil-based liquids, decrease discomfort and increase pleasure during intercourse [6,13,14]. Such lubricants are also used to produce a sensation of ‘wetness’ [6,15], when the body does not produce sufficient natural friction-lowering lubrication (e.g. menopausal and post-menopausal individuals, and individuals experiencing dyspareunia) [16], or for other reasons including enhancing foreplay, curiosity and ‘spicing up’ one's sex life [17,18]. Condom-associated discomfort is a common ‘turn-off’ [7] and a highly cited reason for persuading one's partner to forgo using condoms [19], so many individuals choose to use lubricants to decrease this discomfort. Indeed, of a nationally representative sample of Americans, 61.5% of women and 66.1% of men were found to agree that lubricants make sex feel better [17,18]. The mechanism by which discomfort is relieved and pleasure is increased derives from an interplay between reduction in surface friction and increase in perceptions of gliding, wetness or slipperiness [14]. As a consequence of providing decreased friction, personal lubricants may also extend the duration of intercourse, as many male and female partners mutually believe that longer intercourse is more desirable for a satisfactory sexual intercourse experience [20]. Additional benefits of lubricants include their association with reduced condom breakage [21,22] and slippage [1,23]. There is growing evidence that personal lubricants are becoming a staple of many partners' sexual experiences [14], and thus technology that further improves the sensation of lubrication is highly sought.

However, personal lubricants possess several undesirable properties. A shortcoming of water-based lubricants is that their solubility in physiologic fluids and their proclivity to absorb into bodily tissues limit their extended duration use; [24] likewise, while silicone- and oil-based lubricants exhibit greater robustness from being dissolved in water, these lubricants gradually dissipate from the site of articulation as they are sloughed off at the site of intercourse. A 2008 study of a nationally representative cohort of American adults found that simply the process of putting on a condom was a turn-off [7]. Potentially for this reason, along with others such as convenience, many condoms are packaged with a small amount of silicone lubricant applied to the condom, ostensibly intended to obviate the act of adding extra lubricant. Nonetheless, the perceived quantity of lubricant or magnitude and/or duration of lubrication sensation provided by such pre-lubricated condoms are insufficient for many partners [17,25], and the undesirable step of adding more lubricant is required by these individuals. For these reasons, a method that extends the duration of lubrication without causing inconvenience for the user (i.e. without the need for adding extra lubricant) would improve many condom-users’ sexual experiences and potentially increase condom usage among individuals that do not use condoms due to their real or perceived dissatisfactory lubrication.

**Figure 1.**
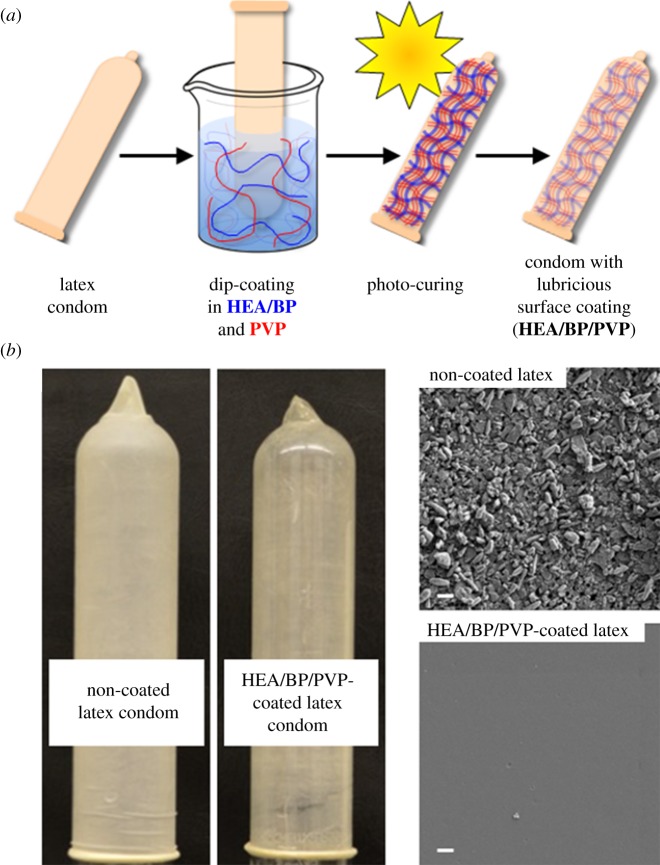
Latex surface modification to afford a hydrophilic, lubricious thin polymer coating. (*a*) The coating scheme is comprised of polymer entrapment of lubricious PVP within macroinitiator HEA/BP, followed by exposure to light activation and chemical cross-linking among HEA/BP, PVP and the latex surface. (*b*) Photographs of non-coated and coated latex condoms. (*c*) Scanning electron micrographs of non-coated and coated latex; scale bar, 1 µm.

To fulfil this unmet need, we developed and optimized a lubricious surface treatment technique involving the coating of natural rubber latex with a thin layer of hydrophilic polymers that, upon contact with water, become slippery to the touch. The strategy renders fluid lubricants unnecessary for delivering a feeling of lubrication, as the latex surface itself is rendered slippery by the surface treatment, and the coating technique extends the duration of lubrication sensation by forming robust covalent bonds between the latex and the thin coating layer (figure 1). We recently reported the synthesis and characterization of hydrophilic two-component coatings, which we demonstrated can be covalently attached onto latex surfaces through a UV-induced reaction to afford increased hydrophilicity. The coatings (abbreviated *HEA/BP/PVP*) comprised (i) photo-macroinitiators composed of 2-hydroxyethylacrylate and benzophone units (HEA/BP) and (ii) hydrophilic polymers (e.g. polyvinylpyrrolidone, PVP) [26]. The frictional properties of the coating, along with the relationship between frictional properties and human touch-derived ‘slipperiness,’ have not been established; furthermore, human preference for a condom containing the coating, and potential future condom use with such a condom, has not to date been surveyed.

## 2. Aims

The purposes of this study are to: (i) assess the frictional performance of HEA/BP/PVP-coated latex compared to non-coated latex and latex lubricated by a personal lubricant, as a function of repeated articulation cycles and presence of physiologic fluid; (ii) evaluate the level of agreement between human-perceived slipperiness and machine-measured friction for latex materials before and after submergence in water, and (iii) ascertain whether survey participants express preference for a hypothetical male condom containing the HEA/BP/PVP coating to determine the potential for such a technology to find epidemiological utility.

## 3. Methods

For complete details for each of the below sections, see electronic supplementary material.

### 3.1. Latex coating procedure

Natural rubber latex sheets were washed, dried and coated as described previously [26]. Briefly, a 1 : 1 water:ethanol solution containing (i) HEA/BP polymer (10 w/w% BP-containing monomer, *M*_n_ 129 kDa as measured via gel permeation chromatography with polystyrene standards) at 5 w/v% and (ii) PVP (*M*_n_ 360 kDa) at 2 w/v% was pipetted (2.0 ml) onto a latex-coated glass slide (3 × 5 inches) which was then exposed to a hand-held UV lamp (365 nm) for 30 min.

### 3.2. Spectroscopic characterization via Fourier transform infrared spectroscopy and contact angle characterization

Fourier transform infrared spectroscopy (FTIR) spectra were obtained for non-coated latex, latex coated with HEA/BP only and latex coated with HEA/BP/PVP [26]. The non-coated latex exhibits no absorbance in the range 1600–1800 cm^−1^; latex coated with HEA/BP only exhibits a strong absorption band at 1731 cm^−1^ (carbonyl stretch of HEA), while latex coated with HEA/BP/PVP displays an additional absorption band at 1655 cm^−1^ (carbonyl stretch).

Contact angle measurements were obtained for non-coated latex, latex coated with HEA/BP only and latex coated with HEA/BP/PVP [26]. Non-coated latex possesses a contact angle of 117.2 ± 5.6°, latex coated with HEA/BP only exhibits a reduced contact angle of 91.5 ± 7.1° and the addition of PVP further reduces the contact angle to 84.8 ± 5.6°.

### 3.3. Friction testing

A friction testing protocol, designed for assessing biologically relevant lubrication, was performed. Briefly, flat latex substrates were brought into contact against a flat, skin-like polyurethane countersurface with one of two lubricants (water or a leading commercially available water-based personal lubricant, KY Liquid^®^) liberally pipetted in between the surfaces prior to initiating contact. The latex samples were loaded at a constant stress (78 kPa for single articulation and 1000-articulation tests, 104 kPa for tests evaluating the effect of large surrounding water volume) and rotated against the countersurface at an effective perimeter velocity of 22 mm s^−1^, while axial normal force and torque were recorded (Electroforce 3200, TA Instruments).

### 3.4. Tensile testing

Rectangular latex strips (1 cm × 8 cm) either not coated or coated with HEA/BP/PVP were soaked in water for approximately 5 s and stretched at a strain rate of 0.5 s^−1^ (5848 Micro-tester, Instron). Tensile stress was converted from the measured tensile force and plotted against tensile strain to derive tensile moduli; a typical plot is shown in electronic supplementary material, figure S1. Latex possesses two types of tensile moduli, referred to as *E_ε_*_<5_ and *E_ε_*_>5_, corresponding to moduli at low and high stretch, respectively. Sample thickness was measured via hand-held callipers.

### 3.5. Leak testing

Coating protocol was scaled up from small rectangular strips to typical male condoms using a dip-coating approach. A non-lubricated male latex condom was fitted onto a penile-shaped glass mandrel and dip-coated, cured, washed and dried. The Water Leak Test as described in ISO 23 409 ‘Annex J: Testing for Holes' was performed: five HEA/BP/PVP-coated condoms were filled with water (300 ml) and checked for leaks by rolling onto coloured absorbent paper as described under the ISO guide.

### 3.6. Latex touch test

An IRB-approved latex touch-test and accompanying survey were administered to a population sample of *N* = 33 participants (13 males and 20 females) of different ages (24–58 yrs), ethnicities, education levels and degrees of sexual activity and condom use (Boston University School of Medicine Protocol # H-33427). Participants were asked to feel and compare three material samples (before and after submergence in and removal from water (to represent physiological fluid during intercourse)): (i) non-coated latex, (ii) non-coated latex lubricated by personal lubricant (100 µl) and (iii) HEA/BP/PVP-coated latex. The order in which the three samples were placed before the participants was randomized for each participant and participants were blinded as to the composition of each sample.

### 3.7. Condom usage and preference survey

Participants were then surveyed regarding frequency of condom use when having sex, preferences for using condoms made from the latex samples they felt during the touch test and preferences for using a condom that were inherently slippery and remained so for a long duration, and whether or not it would make them consider increasing their condom usage.

### 3.8. Statistics

All experiments were performed with a minimum sample size of 3; error bars in figures and ‘±’ notation represent the standard deviation of the mean. Statistically significant differences (95% confidence level, *p* = 0.05) were identified through ANOVA with Tukey–Kramer Multiple Comparisons using a Bonferroni correction for comparisons of continuous variables (coefficient of friction (COF) and slipperiness). Chi tests were used to identify statistically significant majorities (95% confidence level) for comparisons of proportions (latex touch sample material).

## 4. Main outcome measures

### 4.1. Spectroscopic and contact angle characterization

FTIR spectroscopic absorbance energies are reported in cm^−1^, and contact angles of water droplets resting on non-coated or coated surfaces are reported in degrees.

### 4.2. Friction testing

COF was calculated as the ratio of frictional force (converted from measured torque) to measured normal force on the latex. COF values were determined under initial articulation conditions (i.e. over one full rotation of the latex sample) as well as under cyclically repeated articulation to determine cycle- and time-dependence of COF (1000 torsional articulation cycles). COF was also determined in the presence or absence of physiologic fluid.

### 4.3. Touch test and condom usage and preference survey

Participants were asked to first ascribe a slipperiness rating (1, extremely sticky; 7, extremely slippery) to each latex sample before and after submergence in and removal from water, and then to choose how much more slippery the most slippery sample was compared to the others (somewhat, much or very much more slippery). Sexual activity (active or not active) and frequency of condom use (always, usually, occasionally and never) were surveyed. Participants were asked about first-choice preference (or no preference for any one over the others) for a condom containing each sample they touched, as well as preference for various condom types (five-level Likert scale) and whether or not existence of such condoms would cause them to consider increasing their condom usage (five-level Likert scale). Preference for an HEA/BP/PVP-coated condom over existing commercial condoms was surveyed (five-level Likert), as was consideration of using such a condom regularly (five-level Likert).

## 5. Results

### 5.1. Friction testing of HEA/BP/PVP-coated latex samples

#### 5.1.1. Initial articulation conditions

**Figure 2.**
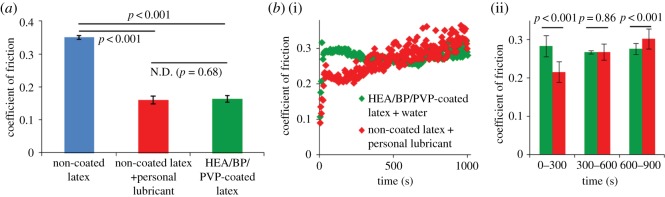
(*a*) COF for non-coated latex lubricated by water, non-coated latex lubricated by personal lubricant and HEA/BP/PVP-articulation (corresponding to 1000 rotations), surrounded by air, for non-coated latex lubricated by personal lubricant and for HEA/BP/PVP-coated latex lubricated by water; *n* = 3, standard deviations represented as error bars. (*b*) COF over 1000 seconds of repeated cyclical articulation (corresponding to 1000 rotations), surrounded by air, for non-coated latex lubricated by personal lubricant and for HEA/BP/PVP-coated latex lubricated by water. (i): friction time courses. (ii): average COFs during early, middle and late segments of the 1000-second test (each segment 300 s in length), revealing the personal lubricant's statistically significantly lower friction during the early segment and HEA/BP/PVP's statistically significantly lower friction during the late segment; *n* = 75 COFs averaged per segment, standard deviations represented as error bars.

Torsional friction testing (deformable polyurethane countersurface rotating against latex samples, 78 kPa stress, effective velocity 22 mm s^−1^) was performed on three sample groups: non-coated latex lubricated by water, non-coated latex lubricated by personal lubricant (KY Liquid^®^) and HEA/BP/PVP-coated latex lubricated by water. HEA/BP/PVP-coated samples demonstrated statistically significantly lower COF (53% lower) than non-coated latex lubricated by water (0.163 ± 0.010 versus 0.350 ± 0.006) and comparable COF compared with non-coated latex lubricated by personal lubricant (0.159 ± 0.012, 55% lower than non-coated latex lubricated by water; figure 2*a*).

#### 5.1.2. Cyclically repeated articulation

Next, friction testing was performed over a 1000-s duration (corresponding to 1000 cycles) to compare the timecourse of lubrication over repeated cyclical articulation occurring for HEA/BP/PVP-coated latex and for non-coated latex lubricated by personal lubricant (figure 2*b*). Initially, lubrication of non-coated latex by personal lubricant exhibits a lower COF than HEA/BP/PVP-coated latex lubricated by water, although the friction provided by the personal lubricant worsens over the duration of the test and ultimately produces higher COFs than the HEA/BP/PVP-coated system by the completion of the test (figure 2*b*(i)). Over the initial 300 s, the COF of non-coated latex lubricated by personal lubricant was statistically significantly lower than that of latex treated with the hydrophilic coating lubricated with water (0.22 compared with 0.28); over the next 300 s, the two groups' COFs were not statistically significantly different (*p* = 0.86), and over seconds 600–900, the COF of non-coated latex lubricated by personal lubricant had increased by over 0.08 to a COF greater than 0.30 (a roughly 40% increase) while the COF for the HEA/BP/PVP-coated sample remained consistent (0.27–0.28) throughout the entirety of the test (figure 2*b*(ii)).

**Figure 3.**
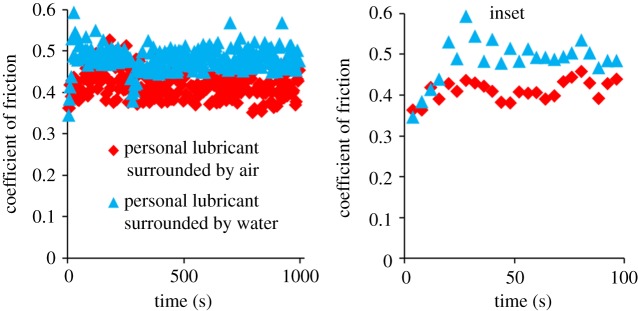
COF over 1000 s for non-coated latex lubricated by personal lubricant, without and with the presence of a surrounding volume of water. COF increases from approximately 0.4 to 0.5 in the presence of the surrounding aqueous fluid.

The same time course friction test was then repeated on non-coated latex lubricated by personal lubricant surrounded by air (as were prior tests) and also on non-coated latex lubricated by personal lubricant but surrounded by a larger volume of water; this configuration involved depositing the lubricant between the latex substrate and polyurethane countersurface, compressing the two materials together, and then adding a surrounding bath of water immediately prior to commencing the cyclical articulations and was performed in order to observe the effect of water's presence on the lubricant's ability to lubricate over a 1000-s duration. The personal lubricant surrounded by air exhibited COFs of approximately 0.4 for nearly the entire test's duration, while the same test performed with a surrounding volume of water was observed to have COFs ca. 25% greater at approximately 0.5 (figure 3). This difference in COF occurs as early as within the first 30 s of the 1000-s test (figure 3, inset).

### 5.2. Tensile testing, leak testing and thickness of HEA/BP/PVP-coated latex samples

HEA/BP/PVP-coated and non-coated rectangular latex strips (1 × 8 cm) were mechanically stretched in tension until failure. The testing revealed similar stress responses (elastic tensile moduli and ultimate tensile stress, UTS) for both non-coated and coated latex specimens when subjected to identical strain profiles; furthermore, male latex condoms that were either non-coated or HEA/BP/PVP-coated both passed leak testing per ISO 23409 (electronic supplementary material; table 1). No statistically significant differences in mechanical parameters or leak testing between non-coated and coated latex were observed; however, thickness statistically significantly increased from 66 ± 2 to 81 ± 2 µm.

**Table 1.** Tensile mechanical parameters, leak testing results and thicknesses for non-coated and coated latex (*N* = 3) and their associated comparative *p*-values.

	*E_ε_*_<5_ (kPa) (low-strain tensile modulus)	*E_ε_*_>5_ (MPa) (high-strain tensile modulus)	UTS (MPa)	leak test result^a^	thickness (µm)
non-coated latex	25.6 ± 6.6	1.69 ± 0.21	10.5 ± 1.4	pass	66 ± 2
HEA/BP/PVP-coated latex	17.2 ± 1.8	1.86 ± 0.10	11.9 ± 4.9	pass	81 ± 2
*p*-value (*t*-test)	0.15	0.36	0.65	n.a.	<0.001^b^

UTS: ultimate tensile stress.

^a^Water leak testing described in detail in ISO 23409 ‘Annex J: Testing for Holes'.

^b^Statistically significant difference.

### 5.3. IRB-approved latex touch test and condom usage and preference survey

#### 5.3.1. Agreement between human touch perception and machine-measured COF

**Figure 4.**
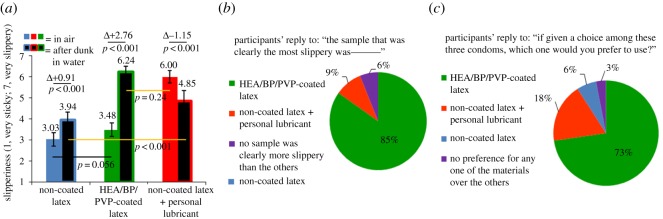
(*a*) Slipperiness ratings, as perceived by touch-test participants, for non-coated latex, non-coated latex lubricated by personal lubricant and HEA/BP/PVP-coated latex both before and after exposure to water. *N* = 33 participants, standard deviations represented as error bars. (*b*) A statistically significant majority (85%) of participants agreed or strongly agreed that latex samples treated with HEA/BP/PVP coating were clearly the most slippery after exposure to water in comparison to the other touch samples; *N* = 33. (*c*) A statistically significant majority (73%) of participants expressed a preference or strong preference for a condom coated with HEA/BP/PVP-coated condom compared to a non-coated condom or non-coated condom lubricated by personal lubricant; *N* = 33.

Touch-test participants (*N* = 33) were asked to rate three samples, both before and after exposure to water, on a 7-point scale, with 7 points representing most slippery and 1 point representing most sticky. The three samples investigated were non-coated latex, non-coated latex lubricated by 100 µl of personal lubricant and HEA/BP/PVP-coated latex. Prior to exposure to water, non-coated latex and HEA/BP/PVP-coated latex possessed similar average slipperiness (3.03 and 3.48, respectively), while latex lubricated by personal lubricant possessed significantly greater average slipperiness (6.00). Following exposure to water, HEA/BP/PVP-coated latex underwent a statistically significant increase in slipperiness of 2.76 points up to 6.24 points, while latex lubricated by personal lubricant underwent a statistically significant decrease in slipperiness of 1.15 points down to 4.85 points (figure 4*a*). After exposure to water, HEA/BP/PVP-coated latex was statistically significantly more slippery than latex lubricated by personal lubricant. When all participants were then asked to choose the most slippery material after water exposure, 85% agreed that latex samples coated with HEA/BP/PVP felt the most slippery (figure 4*b*). Of those that agreed, 70% felt the coating was ‘much’ or ‘very much’ more slippery than the other two (for additional results, see electronic supplementary material).

#### 5.3.2. Relation between condom use, condom material preference and future condom use

Participants were next asked which condom they would prefer if there existed condoms made from each of the three samples they touched during the touch test (non-coated latex, non-coated latex lubricated by personal lubricant and HEA/BP/PVP-coated latex); 73% of participants (a statistically significant majority) chose the condom made from HEA/BP/PVP-coated latex (figure 4*c*).

Participants next were asked about their preferences and potential courses of action if there existed a condom coated with HEA/BP/PVP. Of participants that ‘usually’ or ‘occasionally’ use condoms, 91% agreed they would consider using an HEA/BP/PVP-coated condom regularly, 91% would prefer it over a standard non-lubricated condom, 64% would prefer it over a standard lubricated condom and 55% would consider increasing their condom usage if such a condom were to exist (table 2). Of participants that ‘never’ use condoms, 57% agreed they would consider using an HEA/BP/PVP-coated condom regularly, 71% would prefer it over a standard non-lubricated condom, 50% would prefer it over a standard lubricated condom and 21% would consider increasing their condom usage if the condom existed—i.e. one fifth of participants who never use condoms would consider using them during sex if a condom coated with HEA/BP/PVP were available. When asked not specifically about a condom coated with HEA/BP/PVP but instead about a hypothetical inherently slippery condom, of participants that ‘usually’ or ‘occasionally’ use condoms, 100% would prefer an inherently slippery condom and 82% agreed an inherently slippery condom would increase their condom usage. Of participants that ‘never’ use condoms, 86% would prefer an inherently slippery condom and 43% agreed an inherently slippery condom would increase their condom usage.

**Table 2.** Proportion of participants who agree or strongly agree with statements of opinion on the HEA/BP/PVP coating for condoms and potential courses of action regarding condom usage.

	participant frequency of condom usage during sex →	‘usually’ or ‘occasionally’ (*N* = 11) % (*N*)	‘never’ (*N* = 14) % (*N*)
If there were an HEA/BP/PVP-coated condom on the market, …	…I would consider using it regularly	91 (10)	57 (8)
	…I would prefer it over a standard non-lubricated condom	91 (10)	71 (10)
	…I would prefer it over a standard lubricated condom	64 (7)	50 (7)
	…it would make me consider increasing my condom usage when having sex	55 (6)	21 (3)
	I would prefer an inherently slippery condom	100 (11)	86 (12)
	An inherently slippery condom would increase my usage of condoms when having sex	82 (9)	43 (6)
	I would prefer a condom that inherently stays slippery for a long time	100 (11)	86 (12)
	A condom that inherently stays slippery for a long would increase my usage of condoms when having sex	82 (9)	50 (7)

## 6. Discussion

### 6.1. Selection of coating composition for studies

The lubricating effect of PVP has been reported in the literature [27–29] and is used in several commercial hydrophilic lubricious coatings e.g. those found on a catheter [30–32]. Thus, we developed the present coating strategy as a means of physically entrapping PVP on latex surfaces to render the surface hydrophilic instead of hydrophobic. In pilot studies of coating formulations, concentrations of macroinitiator HEA/BP and of hydrophilic polymer PVP were varied to qualitatively identify coating compositions that were lubricious and also adhered sufficiently to the latex substrate. HEA/BP concentrations above 10 w/v% were too viscous to dissolve homogeneously, and concentrations below 2 w/v% did not yield sufficient radical generation for interpenetration and entrapment of PVP (as evidence by FTIR absorbance of PVP dimishing upon rubbing while HEA/BP's absorbance remained). Thus, a 5 w/v% concentration was selected. PVP concentrations above 5 w/v% yielded a thick lubricious coating that exhibited delamination from the latex substrate, hypothesized to be due to insufficient cohesion within the polymer coating layer and subsequent sloughing off upon rubbing. PVP concentrations below 1 w/v% were not qualitatively lubricious, and thus a 2 w/v% concentration (lubricious to the touch and as measured by quantiative frictional analysis, *vide infra*) was selected.

### 6.2. Friction testing of HEA/BP/PVP-coated latex samples

#### 6.2.1. Initial articulation conditions

Frictional forces during intercourse between latex and mucosal membranes are known to potentially cause detrimental outcomes for partners if not attenuated by endogenous or exogenous lubricant. Discomfort and pain, along with condom breakage, may occur if there is high friction during intercourse, and microtrauma to the mucosal membrane imposed by the high friction may increase the likelihood of STI transmission by exposing direct access to the bloodstream. Thus, we evaluated the ability of HEA/BP/PVP-coated latex to afford a COF lower than non-coated latex or a non-coated latex in the presence of a common commercial water-based personal lubricant. Indeed, HEA/BP/PVP-coated latex lubricated by water exhibited a COF 53% less than that of non-coated latex lubricated by water (figure 2*a*). In comparison, the personal lubricant lowers friction by 55%, so we conclude that the HEA/BP/PVP coating is able to lower friction dramatically and performs similarly to a commercial lubricant in a model where dilution of lubricant does not occur.

#### 6.2.2. Cyclically repeated articulation

In addition to testing frictional performance at initial articulation conditions, we evaluated the HEA/BP/PVP coating's ability to persist, providing low friction over multiple cycles of articulation as well as over a long time duration of testing. As exogenous personal lubricants, which lubricate through a fluid film lubrication mechanism, have the potential for being sloughed off at the site of intercourse, we hypothesize that an inherently lubricious coating, which does not require a fluid film to lower friction, could provide low friction over multiple cycles of friction testing. Repeated testing over 1000 cycles assessed the upper end of physiological relevance, as typical intercourse consists of about 100–500 thrusts [33]. The HEA/BP/PVP coating afforded a relatively invariant COF for the duration of the 1000 cycles. The personal lubricant's COF was initially lower than that of HEA/BP/PVP-coated latex over the first 300 cycles of testing, but the COF rose over time and the friction became statistically significantly greater (i.e. poorer performance) than that provided by the HEA/BP/PVP coating (figure 2*b*). Qualitatively, upon inspection of the sample lubricated by personal lubricant at the completion of the 1000-cycle test, much of the lubricant had been rubbed away and expelled from the articulating surfaces, believed to be the cause of the deterioration in lubricating properties over the course of multiple cycles. By contrast, the HEA/BP/PVP coating is bonded to the latex surface and remains over time.

To further understand the relevant contexts under which the personal lubricant lubricates, we investigated its COF in the presence or absence of a surrounding volume of water. The configuration of personal lubricant in the absence of the water bath (i.e. surrounded by air) represents a physiologically relevant scenario of personal lubricant sloughing off from the articulating areas, while the configuration of personal lubricant surrounded by water represents a physiologically relevant scenario of personal lubricant being diluted by bodily fluids and effectively washed away from the site of articulation. Water imitates the presence and effects of physiological fluids on the latex and tested lubricants during intercourse, and we hypothesized that the water-soluble personal lubricant would gradually dissolve [24] and its lubricating ability would diminish. Indeed, the COF was approximately 25% greater when the articulating surfaces, coated in the personal lubricant, were bathed in a large volume of water (figure 3). The increase in friction occurred in a little as the first 30 cycles after the 1000-cycle friction test began, indicating a decrease in lubriciousness and that an initial sensation of lubrication may not last for a duration suitable for most partners. In comparison, the HEA/BP/PVP-coated latex samples function with low friction irrespective of a large surrounding bath of water, as the lubricating coating is covalently bound to the latex and does not dissolve into the water over time.

### 6.3. Tensile testing, leak testing and thickness of HEA/BP/PVP-coated latex samples

The tensile properties of latex sheets coated with HEA/BP/PVP were measured and compared to non-coated latex samples to investigate whether the coating application protocol (the light exposure, solvent exposure or polymer coating itself) resulted in any deterioration that would weaken the latex material. The testing revealed similar stress responses (tensile modulus and failure stress) for both non-coated and coated latex specimens when subjected to identical strain profiles, indicating that the hydrophilic coating procedure does not weaken the latex material (table 1). Full-size male condoms were coated with HEA/BP/PVP and successfully passed leak testing per condom manufacturing quality controls under ISO 23409; this testing involved filling coated condoms with water, and no leakages were observed, further confirming that the HEA/BP/PVP coating does not weaken the tensile properties or strength of the underlying latex. Upon coating, sample thickness increased by 15 ± 4 µm; this difference is believed to have no discernible effect on condom performance, although future clinical studies of HEA/BP/PVP-coated condoms must address this parameter.

### 6.4. IRB-approved latex touch test and condom usage and preference survey

The touch-test and accompanying survey investigated whether human subjects can distinguish differences in slipperiness among six lubrication scenarios (three latex samples, before and after exposure to water) and to assess the level of agreement between human friction perceptions and mechanical testing machine-determined COFs. The survey further assessed the relationship between participants' perception of latex slipperiness and condom material preference and the implication that their preferences have on their potential future course of action regarding the level of condom use when having sex.

#### 6.4.1. Agreement between human touch perception and machine-measured COF

Non-coated latex felt moderately slippery to participants when dry, and only slightly increased in slipperiness to a rating of 3.94 following exposure to water (7 is very slippery, 1 is very sticky; figure 4*a*). The HEA/BP/PVP-coated latex initially felt similar to non-coated latex when dry, but upon exposure to water, increased in slipperiness rating by 2.76 points to a final rating of 6.24. By contrast, personal lubricant provided the best initial slipperiness (6.00 rating) but decreased in slipperiness to 4.85 following exposure to water. This comparison highlights that the HEA/BP/PVP coating was statistically significantly more slippery than the personal lubricant when both samples were exposed to water (in fact 85% of participants agreed the HEA/BP/PVP-coated latex was the most slippery of all three samples; figure 4*b*), and that even before the personal lubricant sample was exposed to water, the HEA/BP/PVP-coated sample is slightly more slippery (6.24 versus 6.00 rating, *p* = 0.24) albeit not statistically significantly. Furthermore, the results from the direct human perception of slipperiness are in good agreement with machine-measured COFs, as the slipperiness/lubrication provided by the HEA/BP/PVP coating and personal lubricant is approximately equivalent as measured both by human touch as well as machine, and slipperiness decreased (COF increased) following exposure of the personal lubricant to water.

**Figure 5.**
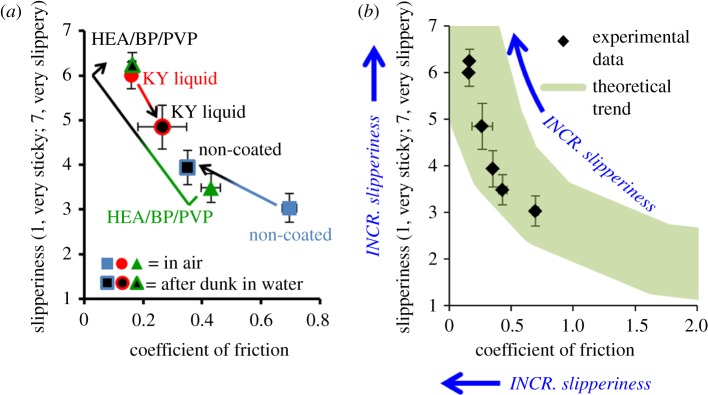
(*a*) Correlation between machine-measured COF and human-perceived slipperiness for three samples, prior to after dunking in water. (*b*) Overlay of experimentally gathered data with the theoretical trend for the correlation between COF and perceived slipperiness. *N* = 33, standard deviations represented as error bars.

The agreement between friction coefficients and human perception of slipperiness has been given recent attention in the literature surrounding tactile materials science (studies of plastic, glass and paper-based materials) [34,35], but it has not been extensively studied in the sexuality psychoanalysis field. This agreement is visualized by plotting the direct correlation between COF and human-perceived slipperiness (linear, *R*^2^ = 0.83, two-tailed *p* = 0.011; figure 5*a*). Both non-coated latex and HEA/BP/PVP-coated latex moved towards the upper-left quadrant of the correlation plot (increased in slipperiness) following exposure to water, whereas non-coated latex lubricated by personal lubricant shifted towards the lower-right quadrant of the plot (decreased in slipperiness) following exposure to water. The six-point correlation observed between human-perceived and machine-measured slipperiness agrees with the theoretical trend projected in figure 5*b*. While a dataset of limited sample size was collected (six points), the statistically significant correlation indicates that machine-measured friction coefficients in the range approximately 0.1–0.7 reflect the slipperiness detected by human fingers.

#### 6.4.2. Relation between condom use, condom material preference and future condom use

Following the touch test (which kept participants blinded as to the intended application of the latex materials), the electronic survey then asked them their level of condom use when having sex (always, usually, occasionally or never), and next asked which condom they would prefer if there existed condoms made from each of the three samples they touched during the touch test; 73% of participants chose the condom coated with HEA/BP/PVP, followed by 18% choosing a condom with personal lubricant (figure 4*c*). This statistically significant majority of participants that expressed a preference for an HEA/BP/PVP-coated condom indicates the potential adoption of such a condom in the broader population of condom users. Participants were then asked to consider the existence of an HEA/BP/PVP-coated condom available for use, and a statistically significant majority of participants that ‘usually’ or ‘occasionally’ use condoms either ‘agreed’ or ‘strongly agreed’ that they would consider using the condom regularly and that they would prefer it over a standard non-lubricated condom (table 2). Many participants, including those that ‘never’ use condoms, expressed a preference for the HEA/BP/PVP-coated condom over a standard *lubricated* condom and agreed that it would make them consider increasing their condom usage when having sex. When participants were asked about a hypothetical condom that is ‘inherently slippery’ and ‘inherently stays slippery for a long time,’ usual and occasional condom users, and even many non-condom users, expressed a preference for such condoms and agreed they would increase their condom usage if such condoms existed.

#### 6.4.3. Limitations

The number of participants in the touch-test and survey (33) warranted sufficient statistical power to identify significant differences among the three latex samples, as well as between samples before and after submergence in water. However, these findings will need to be confirmed in a larger study of user preference following touch-testing. Moreover, participants were asked prospectively about their opinions regarding HEA/BP/PVP-coated latex condoms, without having used such condoms for intercourse (as such condoms are not currently available on the market). Prior to any clinical study in which participants use HEA/BP/PVP-coated condoms for intercourse, FDA approval must first be obtained.

An additional limitation of this study is that the touch-test conducted did not incorporate a non-coated latex sample lubricated by silicone-based personal lubricant (e.g. that found on the majority of latex male condoms packaged with lubricant). It is known that silicone personal lubricants are more resistant to being diluted and washed away in the presence of aqueous fluids; however, silicone lubricants nonetheless slough away from the site of intercourse with time; a future study comparing the HEA/BP/PVP coating to silicone lubricants should thus incorporate an element of repeating rubbing to assess the human-perceived duration of slipperiness.

## 7. Conclusion

In summary, we document the friction-lowering properties of an HEA/BP/PVP-based novel coating for latex. The coating does not affect the strength of the latex, and the coating provides consistently low friction even when subjected to large volumes of water or 1000 cycles of articulation. In contrast to the novel coating, the lubricating capabilities of a leading water-based personal lubricant, which functions via the formation of a lubricant fluid film at the latex interface worsens as a function of repeated rubbing cycles, as well as exposure to water. Finally, an IRB-approved touch test and condom material preference survey reveals that humans perceive latex slipperiness in a manner that agrees with machine-measured COFs and that participants displayed a preference for the HEA/BP/PVP-coated latex. The survey results suggest that an inherently slippery condom could be adopted and could increase condom usage among populations that do not consistently use condoms. Taken in conjunction with the results of the condom preference survey, as our mechanical testing data indicate that the HEA/BP/PVP coating confers a lubricious surface property to latex that remains consistently slippery for a long duration, an HEA/BP/PVP-coated condom shows potential to be an effective strategy for increasing condom usage among populations with a high incidence of STI transmission [9,10,36,37] and unplanned pregnancy [38–41].

## Supplementary Material

Supporting Informaton;Survey results

## Data accessibility

Details of mechanical testing and survey of slipperiness and condom use preferences. This material is available as electronic supplementary material.
