# Supplementary material for: Friction-lowering capabilities and human subject preferences for a hydrophilic surface coating on latex substrates: implications for increasing condom usage
Source: R Soc Open Sci. 2018 Oct 17;5(10):180291. doi: 10.1098/rsos.180291 (PMC6227966; doi:10.1098/rsos.180291)
Supplement: Supporting Informaton;Survey results [file rsos180291supp1.docx]

{Derouet, 2009 #4}Supporting Information for:

“Friction-lowering Capabilities and Human Subject Preferences for a Hydrophilic Surface Coating on Latex Substrates: Implications for Increasing Condom Usage”

Benjamin G. Cooper, PhD,^1^ Stacy L. Chin, PhD,^1^ Ruiqing Xiao, MS,^1^ Karen Buch, MD,^2^ Ducksoo Kim, MD,^2^ Mark W. Grinstaff, PhD*^1,3,4^

Author affiliations:

^1^Department of Chemistry, Boston University, Boston, Massachusetts 02215, United States

^2^Department of Radiology, Boston University School of Medicine, Boston, MA 02118, United States

^3^Department of Biomedical Engineering, Boston University, Boston, Massachusetts 02215, United States

^4^Department of Medicine, Boston University School of Medicine, Boston, MA 02118, United States

*Corresponding author: Mark W. Grinstaff, PhD, Departments of Chemistry, Biomedical Engineering, and Medicine, Boston University, Boston, MA 02215, USA. Tel: 617-358-3429; E-mail: mgrin@bu.edu.

Contents Page

**Methods S2**

General methods, materials, and instrumentation S2

Latex coating procedure S2

Scanning electron microscopy S2

Friction testing S2

Tensile testing S3

Leak testing S3

Latex touch-test S3

Condom usage and preference survey S7

Statistics S9

**Supplementary Results and Conclusions S9**

Tensile testing: typical stress-strain plot S9

Figure S1

Tensile testing: typical fatigue test plots S10

Figure S2

Latex touch-test S11

Figures S3-S6

Condom usage and preference survey S13

Figures S7-S9, Table S1

**Supplementary References S15**

**Methods**

**General methods, materials, and instrumentation**. Materials and chemicals were purchased from Sigma Aldrich (St. Louis, MO) and immediately used as received unless otherwise noted. Reactions were carried out using standard techniques.

**Latex coating procedure.** Natural rubber latex sheets were washed, dried and coated as described in a prior report.^1^ Briefly, latex sheets were washed and dried with water and ethanol and mounted onto glass slides. A solution was prepared with 5 w/v% HEA/BP macroinitiator (MW 100,000 Da via GPC with 10 w/w% BP units to HEA units) and 2 w/v% PVP and mixed in an ethanol/water solution (1/1 v/v) until homogeneous. A thin layer of HEA/BP/PVP was applied to coat the surface of the latex sheet and was exposed to UV light (365 nm) for 10 minutes. Coated latex samples were washed with water and ethanol to remove excess non-bonded polymer and dried overnight at room temperature before subjecting the coated latex samples to mechanial testing.

**Fourier transform infrared spectroscopy.** Infrared spectroscopy was performed on a Nicolet FT-IR (Thermo Scientific, Waltham, MA) in attenuated total reflection mode with a resolution of 0.25 cm^-1^ and number of scans = 32.

**Contact angle characterization.** A Kruss DSA 100 contact angle goniometer was used along with deionized water droplets (4 μL) to characterize hydrophilicty of coated and uncoated NR in air. Angles were calculated using the sessile drop fit method on distinct locations on coated samples. Contact angle measurements were performed using frame-by-frame using the Drop Shape Analysis software provided by Kruss and a hanging drop method was used to apply the water droplets on the samples.

**Scanning electron microscopy.** Microscopy was performed at 0 °C and magnification 1 K X and 5 K X, with EHT 2 kV, and aperture size 30 *µ*m.

**Friction testing.** A friction testing protocol, designed for assessing biologically-relevant lubrication, was performed. Latex was adhered via cyanoacrylate glue to a 12-mm diameter ultra high molecular weight polyethylene flat cylindrical substrate. The polyethylene cylinder was placed into a fixture mounted to the servomotor of a TA Instruments Electroforce 3200 dynamic mechanical analysis instrument. Lubricant (either water or a leading commercially available aqueous personal lubricant containing water, glycerin, sorbitol, propropylene glycol, hydroxyethylcellulose, benzoic acid, methyl paraben, and sodium hydroxide) was introduced to the articulating surfaces either by pipetting liberal volume on top of the latex before compressing a flat polyurethane countersurface against it (friction test conducted “surrounded by air”) or by placing a large volume of water surrounding the entire latex sample and the surface of the polyurethane in the lubricant to ensure continued water presence (friction test conducted “surrounded by water”). A 7-mm diameter polyurethane cylindrical specimen (McMaster-Carr, durometer hardness of 40A, “medium soft”) was selected as the articulating counter-surface due to its tissue-like conformability. The polyurethane specimen was loaded axially into a fixture mounted to a load and torque cell, and a normal pressure between the latex and polyurethane specimen of 78 kPa was obtained by controlling the displacement of the polyurethane countersurface. Upon establishing the desired normal force, the latex surface was rotated against the polyurethane countersurface at an angular velocity of 2π rad/sec, corresponding to an effective velocity at the sample’s perimeter of 22 mm/s.

**Tensile testing.** Rectangular latex strips (1 cm x 8 cm) either not coated or coated with hydrophilic polymer as described previously, were soaked in water for approximately 5 seconds and mounted with clamps on a tensile testing apparatus (5848 Micro-tester, Instron). The samples were stretched at a strain rate of 0.5/sec, while a load cell recorded the measured tensile force which was converted to tensile stress using the cross sectional area of the latex strips (determined via calipers). Tensile stress was plotted against tensile strain (change in initial length divided by initial length) to derive tensile moduli; a typical plot is shown in Figure S1. Tensile testing of latex typically reveals two linear regions in the stress-strain curve: a low-strain tensile modulus, *E*_ε<5_, corresponding to the elastic modulus for strain values less than 5, and a high-strain tensile modulus, *E*_ε>5_, that engages typically when tensile strain reaches about 6-10, immediately prior to tensile failure.

Fatigue tensile testing was also conducted. Latex strips prepared as in the previous paragraph were cyclically stretched and released at a frequency of 1 Hz (half second stretching, half second releasing). Tensile strain oscillated between 0 (taut, non-stretched) and 2 (tripled in length), while tensile force was measured and converted to tensile stress as above. Fatigue testing was performed for 500 cycles, as typical intercourse is reported as consisting of 100-500 thrusts.^2^

**Leak testing.** In anticipation of requirements in place for manufacturing under GMP standards, coating protocol was scaled up to apply the hydrophilic coating to male latex condoms using a dip-coating approach. Non-lubricated male latex condoms (Durex^®^) were unrolled, washed, and dried before fitting onto a penile-shaped glass mandrel (diameter of 4 cm and length of 20 cm). The condom was dipped into a solution containing 5% (w/v) HEA/BP and 2% (w/v) PVP and carefully raised to obtain a thin and even layer of the solution coated on the entire condom surface. This mold was immediately placed onto an apparatus that vertically rotated the coated latex condom at 45 rpm while being exposed to UV light for 25 minutes. The coated latex sample was washed in a water/ethanol mixture and dried under a steady airflow at room temperature. Through this application approach, a thin, even, and stable coating resulted on the condom surface even after the washes and rubbing the coated latex condom with fingers in the presence of water. To determine whether the coating application or UV exposure resulted in any visual defects or holes inflicted to the latex, the Water Leak Test as described in ISO 23409 “Annex J: Testing for Holes” was performed. Five male latex condoms were prepared and treated with the hydrophilic coating. The latex condoms were removed from the glass mold and mounted onto an apparatus at the open end to allow 300 mL of water to fill the condom while being suspended in the air at 25 °C. All five coated condoms tested did not show any signs of visible leakage to two observers when tied and rolled onto colored absorbent paper as described under the ISO guide.

**Latex touch-test.** A latex touch-test and accompanying survey were administered to a population sample of thirty three participants (13 males and 20 females) of different ages (24-58 yrs), ethnicities, education levels, and degrees of sexual activity and condom use. The touch-test and survey were approved by the Boston University School of Medicine Institutional Review Board (IRB) (Protocol # H-33427).

Summary: Participants were asked to feel and compare three material samples (before and after submergence in and removal from water (to represent physiological fluid during intercourse): 1) non-coated latex, 2) non-coated latex lubricated by personal lubricant (100 *µ*L), and 3) HEA/BP/PVP-coated latex. The order in which the three samples were placed before the participants was randomized for each participant. Participants were blinded as to the composition of each of the three samples, and were asked electronically (via tablet interface) to rate each sample on a 7-point scale, with 7 points representing most slippery and 1 point representing most sticky.

Detailed description: Touch-test exclusion criteria included having a latex allergy and being pregnant or possibly pregnant. Participants were informed prior to test initiation that the test is composed of “a touch-test followed by some personal questions” and that “participation is voluntary, responses are completely anonymous, and the test can be stopped at any time.” Participants were offered candy in exchange, The touch-test and survey were administered in a neutral-color and minimally decorated room containing a table, chairs for the survey administrator and survey participant, a tablet for the participant to record answers via a self-guided questionnaire, and three latex samples (strips). The three latex samples are each on a glass slide, and are arranged horizontally in front of the participant (A, B, and C, reading left to right). Also on the table are a beaker of water (for the water submergence portion of the test), a clear unmarked glass vial of personal lubricant (KY Liquid^®^ brand), a pipettor for measuring the 100 *µ*L volume of lubricant, and paper towels. Before each participant entered the test room, the administrator placed the three samples on the table, pippetted the lubricant onto the appropriate sample, and opened a new questionnaire on the tablet.

Test initiation: Participant walks into room. Survey administrator says:

*Hello - I’ll be your survey administrator, and I’d like to thank you for volunteering to participate in our survey, which should take about 10 minutes to complete.  This survey is part of a research study, and we must inform you that you’re under no obligation to participate, and you can stop participating at any time.  The purpose of this survey is to learn about people’s sense of touch, specifically slipperiness, and your participation will involve touching several materials, and answering a few questions about them. If you have any questions about this survey, you can contact our study coordinator, Dr. Karen Buch, and I can give you her contact information at the end of the survey if you’d like..*

*In front of you, you’ll see that there are 3 glass slides, each with a thin material sample lying flat on top of the glass surface.   Before you begin feeling them, I’ll read you some instructions first.  Using any combination of pointer finger, middle finger, and ring finger fingertips on your dominant hand, please stroke the samples, one at a time, in any way that feels comfortable and natural. Please stroke all three in a similar manner, because you’ll be asked to compare their slipperiness. Two of the samples are dry to the touch, and you can go back and forth between them to feel them;  one sample contains a drop of liquid that will be on your fingertips after you’ve touched it, so if you’d like to go back to touch the other two once you’ve begun touching* [Administrator points to appropriate sample] *this one, you’ll have to rinse and dry your fingers before returning to the other two].*

*At this time, please go ahead and feel all three samples, and then you can begin answering the questions on this tablet, and you can continue to feel them as you answer questions.  When it asks you to pause, please let me know.*

Test procedure and questionnaire regarding touch-test: The participant then touches the three samples and begins answering questions on the tablet. Questions are below.

*Do you have a latex allergy?* Response choices: “Yes” or “No.”

*1. Please rate each sample (A, B, and C) on the following scale:* Response choices for each of “A,” “B,” and “C”: “Very slippery,” “Slippery,” “Somewhat slippery,” “Neither slippery not sticky,” “Somewhat sticky,” “Sticky,” or “Very sticky.”

*2. For each of the two following questions, please select one of the four choices presented.*

*The sample that was clearly the least slippery was:* Response choices: “Sample A,” “Sample B,” “Sample C,” or “none of the samples was clearly less slippery than the others / two or three of the samples were tied for least slippery”.

*The sample that was clearly the most slippery was:* Response choices: “Sample A,” “Sample B,” “Sample C,” or “none of the samples was clearly more slippery than the others / two or three of the samples were tied for most slippery”.

If the participant indicated that one sample was more slippery than the others, question 2a and its preceding text appeared. If the participant did not indicate one sample was more slippery than the others, question 2a and its preceding text did not appear.

*You indicated one sample was clearly more slippery than the others.*

*2a. Please fill in the blank. The most slippery sample was ______ more slippery than the others.* Response choices: “Slightly,” “Somewhat,” “Much,” or “Very much.”

*Please pause here and inform the survey administrator that you are finished with this section.*

Survey administrator says:

*Now, I’ll take the same three samples, and ask you to repeat the same touch evaluation, after the samples have been dunked in water.* [Instructor shows water.] *In this phase of the survey, it is fine to go back and forth among the three samples to feel them, and you can dunk your fingers in the water to re-wet them if the samples start to dry out.  I’ll reread the brief instructions that I read before, which are:  Using any combination of pointer finger, middle finger, and ring finger fingertips on your dominant hand, please stroke the samples, one at a time, in any way that feels comfortable and natural. Please stroke all three in a similar manner, because you’ll be asked to compare their slipperiness. Please go ahead and feel all three samples, and then you can begin answering the questions on this tablet, and you can continue to feel them as you answer questions.  When it asks you to pause, please let me know.*

Survey Administrator dunks each sample in water for approximately five seconds and returns sample to in front of the participant. The participant then touches the three samples and answers questions on the tablet. Questions are below.

*1’. Please rate each sample (A, B, and C) on the following scale:* Response choices for each of “A,” “B,” and “C”: “Very slippery,” “Slippery,” “Somewhat slippery,” “Neither slippery not sticky,” “Somewhat sticky,” “Sticky,” or “Very sticky.”

*2’. For each of the two following questions, please select one of the four choices presented.*

*The sample that was clearly the least slippery was:* Response choices: “Sample A,” “Sample B,” “Sample C,” or “none of the samples was clearly less slippery than the others / two or three of the samples were tied for least slippery”.

*The sample that was clearly the most slippery was:* Response choices: “Sample A,” “Sample B,” “Sample C,” or “none of the samples was clearly more slippery than the others / two or three of the samples were tied for most slippery”.

If the participant indicated that one sample was more slippery than the others, question 2a’ and its preceding text appeared. If the participant did not indicate one sample was more slippery than the others, question 2a’ and its preceding text did not appear.

*You indicated one sample was clearly more slippery than the others.*

*2a’. Please fill in the blank. The most slippery sample was ______ more slippery than the others.* Response choices: “Slightly,” “Somewhat,” “Much,” or “Very much.”

*Please pause here and inform the survey administrator that you are finished with this section.*

Survey administrator says:

*This concludes the touch-test portion of the survey.  We’d now like to ask you to answer just a few more questions on the tablet.  Please read all questions carefully--for example, sometimes there’s a statement, followed by a question--please do make sure to read everything that’s there.*[Administrator stands up and begins stepping away] *I’m going to step away to give you some privacy; you can go ahead and click continue on the survey, and please let me know once you’re finished.*

Participant then takes remainder of survey while administrator is seated at another table in the room, out of view of the tablet screen, and facing away from the participant.

**Condom usage and preference survey.**

Summary: Following the touch test, participants were then asked to fill out a survey on the tablet regarding their frequency of condom use when having sex, their preferences for using condoms made from the latex samples they felt during the touch test, and their preferences for using a condom that inherently remained slippery and whether or not it would make them consider increasing their condom usage.

Detailed description: The survey began immediately following the touch-test, once the administrator stepped away from the participant to allow privacy while completing the survey. The entirety of the survey is self-guided through the tablet interface. The three samples (A-C after being submerged in water) were still present on the table in front of the participant so that he or she could visualize the samples while answering the survey. Survey instructions and questions are below.

*Follow-up questions.*

*The samples you touched during the touch-test were latex. Latex is a material commonly used in the manufacture of male condoms.*

*We will now ask you some questions about your condom use and preferences.*

*Please remember that all responses are completely anonymous, and you may stop participating at any time.*

*In the last year, have you been sexually active?* Response choices: “Yes” or “No.”

If participant responded “Yes,” then they were asked the following question about their condom use during the 10 times they had intercourse. If they responded “No,” then the questions about their condom use were not administered, and they were next shown text reading “For the remaining questions in the survey, please consider how you would answer the questions if you were to use condoms,” and the next question that appeared was related to Samples A, B, and C and their hypothetical use as condom materials.

*Thinking about the last 10 times you had sex, how much of the time did you and your partner use a condom?* Response choices: “Never,” “Occasionally,” “Usually,” or “Always.”

If the participant responded with “Never,” “Occasionally,” or “Usually,” then the following question with preceding text appeared. If the participant responded with “Always,” then the following question and its preceding text did not appear.

*You indicated that at least some of the time, you have had sex without using a condom.*

*What was the reason for not using a condom? Please check all that apply.* Response choices were: “I / my partner used another method of birth control,” “I don’t / my partner doesn’t like the way condoms feel,” “My partner and I were trying to get pregnant,” and “Other: ________,” for which the participant was allowed to type a reason. This question was optional, and could be skipped if so desired.

*Please think about Samples A, B, and C after they were dunked in water.*

*3. If given a choice among three condoms, one being made from Sample A, another being made from Sample B, and another being made from Sample C, which one would you prefer to use?* Response choices: “Sample A,” “Sample B,” “Sample C,” or “No preference for any one of the materials over the others.”

*This series of questions is related to Sample* [“A,” “B,” or “C” appeared here, corresponding to the HEA/BP/PVP-coated sample, still located in front of the participant to visualize]*, after it was dunked in water.*

*4. For the following statements, please indicate your level of agreement or disagreement. "If there were a condom on the market made from Sample* [the same letter (“A,” “B,” or “C” as above]*...*

*...I would consider using it regularly."* Response choices: “Strongly agree,” “Agree,” Neither agree nor disagree (or N/A),” “Disagree,” or “Strongly disagree.”

*...I would prefer it over a standard non-lubricated condom."* Response choices: “Strongly agree,” “Agree,” Neither agree nor disagree (or N/A),” “Disagree,” or “Strongly disagree.”

*...I would prefer it over a standard lubricated condom."* Response choices: “Strongly agree,” “Agree,” Neither agree nor disagree (or N/A),” “Disagree,” or “Strongly disagree.”

*...it would make me consider increasing my condom usage when having sex."* Response choices: “Strongly agree,” “Agree,” Neither agree nor disagree (or N/A),” “Disagree,” or “Strongly disagree.”

*Gel or liquid lubricants, sometimes called personal lubricants, are products on the market that are used by some individuals. These gel or liquid lubricants are typically either applied by the individuals using them or are included in many varieties of individually wrapped condoms.*

*5. For the following statements, please indicate your level of agreement or disagreement:*

*"I would prefer a condom that is inherently slippery without needing to have any additional gel or liquid lubricant present."* Response choices: “Strongly agree,” “Agree,” Neither agree nor disagree (or N/A),” “Disagree,” or “Strongly disagree.”

*"A condom that is inherently slippery would increase my usage of condoms when having sex."* Response choices: “Strongly agree,” “Agree,” Neither agree nor disagree (or N/A),” “Disagree,” or “Strongly disagree.”

*Gel or liquid personal lubricants can lose their lubricating ability over time as sexual activity proceeds.*

*6. For the following statements, please indicate your level of agreement or disagreement:*

*"I would prefer a condom that inherently stays slippery for a long time without needing to apply/reapply any additional gel or liquid lubricant."* Response choices: “Strongly agree,” “Agree,” Neither agree nor disagree (or N/A),” “Disagree,” or “Strongly disagree.”

*"A condom that inherently stays slippery for a long time would increase my usage of condoms when having sex."* Response choices: “Strongly agree,” “Agree,” Neither agree nor disagree (or N/A),” “Disagree,” or “Strongly disagree.”

*Physiologically, what is your sex?* Response choices: “Male,” “Female,” or “Other.”

*Thank you! This concludes the survey. We greatly appreciate your participation. Please contact study coordinator Dr. Karen Buch if you have any questions about this survey.*

*At this time, please click "submit" and inform the survey administrator that you have finished.*

Upon participant stating they have completed the survey, they’re offered a selection of bite-size candy bars, thanked again, and dismissed.

**Statistics.** All experiments were performed with a minimum n value of 3; error bars in figures and “±” notation represent the standard deviation of the mean, unless otherwise noted. Statistically significant differences (95% confidence level, p = 0.05) were identified through ANOVA with Tukey-Kramer Multiple Comparisons using a Bonferroni correction for comparisons of continuous variables (coefficient of friction and slipperiness). Chi tests were used to identify statistically significant majorities (95% confidence level) for comparisons of proportions (latex touch sample material) as well as for proportion of individuals who agreed with statements (condom use and preference survey).

**Supplementary Results and Conclusions**

**Tensile testing: typical stress-strain plot.** Low-strain elastic modulus *E*_ε<5_ and high-strain elastic modulus *E*_ε>5_ are observed in a typical stress-strain plot (**Figure S1**).

**Figure S1.** Tensile stress is plotted vs tensile strain for a typical latex strip undergoing tensile testing at strain rate of 0.5/sec.

**Tensile testing: typical fatigue test plots.** Both non-coated and HEA/BP/PVP-coated latex undergo 500 cycles of tensile expansion and release with minimal change in tensile stress by the completion of the test compared to the tensile stress at the beginning of the test (**Figure S2**).

**Figure S2.** Tensile stress as a function of time for samples tested under repeated tensile cycling (fatigue). Data displayed for initial 50 seconds, and for 10-second intervals at 100, 200, 300, and 400 seconds into the test, as well as for the final 10 seconds.

**Latex touch-test.** Raw touch-test and survey results are included as a separate Microsoft Excel spreadsheet. Select results are presented and discussed below. “Non-lubricated” corresponds to “non-coated latex” in the manuscript, “hydrophilic coating” corresponds to “HEA/BP/PVP-coated latex” in the manuscript, and “KY Liquid” corresponds to “non-coated latex + personal lubricant” in the manuscript. It should be noted that participants did not see these descriptive names; the names are only used for data analysis purposes.

When the samples were presented initially to the participants, roughly equal groups of participants believed that the non-lubricated sample and the sample with hydrophilic coating were the least slippery sample. Indeed, both of these two samples received similar low slipperiness numerical ratings (3-3.5 on our slipperiness rating scale). Participants overwhelmingly believed the sample lubricated by KL Liquid was the most slippery, agreeing with the average slipperiness rating of 6. Majority was determined statistically significant via chi-squared test (statistic value < 0.0001).

**Figure S3.** Participants’ responses to: “2. The sample that was clearly the least slippery was ______” (left) and “2. The sample that was clearly the most slippery was _______” (right); N=33.

The qualitative magnitude by which the KY Liquid sample was more slippery than the others was more so strong than it was weak, with 87% of participants agreeing it was “much” or “very much” more slippery than the other samples.

**Figure S4.** Of the N=32 participants (97%) that chose KY Liquid as “clearly the most slippery” in question 2, participants’ response to "The most slippery sample was _____ more slippery than the others."

After the samples were all submerged in water, perceptions of slipperiness changed. The sample perceived least slippery was the non-lubricated latex sample, and indeed, its slipperiness rating was the lowest of the three following submergence in water. The sample perceived most slippery was that coated with the hydrophilic coating, also possessing the highest slipperiness rating. Majority was determined statistically significant via chi-squared test (statistic value < 0.0001).

**Figure S5.** Participants’ responses to: “2’. The sample that was clearly the least slippery was ______” (left) and “2’. The sample that was clearly the most slippery was _______” (right); N=33. Right-hand graph data identical to Figure 4b in manuscript.

Participants felt the magnitude of more slipperiness of the hydrophilic coating generally ranged from somewhat more slippery to very much more slippery (68% agreed it was “much” or “very much” more slippery than the others).

**Figure S6.** Of the N=28 participants (85%) that chose the hydrophilic coating as “clearly the most slippery” in question 2’, participants’ response to "The most slippery sample was _____ more slippery than the others."

**Condom usage and preference survey.**

Participant demographics with respect to sexual activity within the last year, and each individual’s sex, were obtained. A roughly equal number of male and female participants were sought, to better represent the wider population of heterosexual sexual partners; approximately a 60/40 F/M ratio comprised the respondent pool, rather than 50/50.

**Figure S7.** Participants’ responses to: “In the last year, have you been sexually active?” (left) and “Physiologically, what is your sex?” (right); N=33.

Frequency of condom use was surveyed for several reasons. First, as reasons for lack of condom use have been surveyed and analyzed in prior reports,^3^ we sought to validate that there was good agreement between former studies and the present study with respect to reasons for forgoing condoms; indeed, this general agreement exists. Second, the authors were particularly interested in the effect of an inherently lubricious condom on participants who usually or occasionally use condoms, as this group of users may be influenced by factors such as pleasure to consider increasing their condom usage—a consideration generally regarded as favorable by many major healthcare institutions. Individuals who never use condoms were hypothesized to be less open to considering the use of a new inherently lubricious condom; however, that some of the non-condom users responded with consideration of using such a condom indicates that even among the group of users thought to be resistant to condom use, there is the potential to increase condom use even among these individuals.

**Figure S8.** Participants’ responses to: “Thinking about the last 10 times you had sex, how much of the time did you and your partner use a condom?”; N=33.

The primary reason for not using condoms was that an alternate method of birth control was being used. However, this question only ascertains a superficial reason, as the clear follow-up question, were the survey instead conducted in live-interview style, would be to inquire why the alternate method of birth control is used instead of condoms. (E.g. due to condoms being less pleasurable than no condom, or due to other desirable characteristics of hormonal birth control methods, for instance.)

**Figure S9.** Participants’ who were sexually active and did not “always” use a condom responses to: What was the reason for not using a condom all of the time? One of the N=25 individuals did not provide a reason, while N=24 provided at least one reason. The N=24 responding participants providing a total of 28 responses (four individuals provided two reasons, while all remaining individuals provided one reason).

**Table S1.** Participants expressing agreement (“agree” or “strongly agree”) with the statements in questions 4, 5, and 6, stratified by frequency of condom use when having sex. Each cell provides: number of individuals, percentage of individuals, and chi-squared statistic based on a null hypothesis of each option on the five-level Likert scale having equal probability of being selected. Chi-squared statistic values < 0.05 are displayed as green, while those > 0.05 are displayed as red. For each question, if the four chi-squared values in that question’s column are all either green or red, the question text is displayed accordingly colored; if a question has both green and red chi-squared statistic values in that question’s column, then the text is displayed black. Rows highlighted yellow indicate identical data to that presented in the manuscript.

**
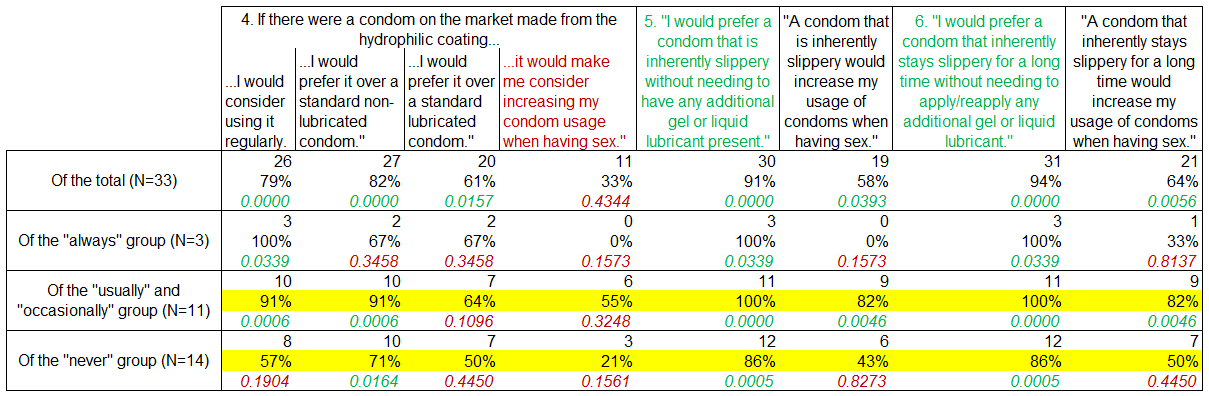
**

An additional striation of agreement with questions 4-6 involves specifically evaluating answers for the individuals who states that they do not condoms all of the time due to reasons of not liking the feeling of condoms. Of the N=9 participants expressing such feelings, 7 participants (78%) either agreed or strongly agreed that A) a condom made from the hydrophilic coating would make them consider increasing their condom usage when having sex, B) an inherently slippery condom would increase their usage of condoms when having sex, and C) a condom that inherently stayed slippery for a long time would increase their usage of condoms when having sex.

**Supplementary References**

[1] Chin SL, Xiao RQ, Cooper BG, et al. Macromolecular photoinitiators enhance the hydrophilicity and lubricity of natural rubber. *Journal of Applied Polymer Science*. 2016;**133**.

[2] Hardy SB, Whitten PL. Patterning of sexual activity. In: Smus BB, Cheney DL, Seyfarth RM, Wrangham RW, Struhsaker TT, eds. *Primate Societies*. Chicago: Chicago University Press; 1987:370-84.

[3] Herbenick D, Schick V, Reece M, et al. Characteristics of Condom and Lubricant Use among a Nationally Representative Probability Sample of Adults Ages 18-59 in the United States. *Journal of Sexual Medicine*. 2013;**10**: 474-83.
